# Crystal structure of 5-(4,5-di­hydro-1*H*-imidazol-2-yl)-3-methyl-1-phenyl-1*H*-pyrazolo­[3,4-*b*]pyrazin-6-amine

**DOI:** 10.1107/S160053681402354X

**Published:** 2014-10-31

**Authors:** Joel T. Mague, Shaaban K. Mohamed, Mehmet Akkurt, Talaat I. El-Emary, Mustafa R. Albayati

**Affiliations:** a Department of Chemistry, Tulane University, New Orleans, LA 70118, USA; b Chemistry and Environmental Division, Manchester Metropolitan University, Manchester M1 5GD, England; c Chemistry Department, Faculty of Science, Minia University, 61519 El-Minia, Egypt; d Department of Physics, Faculty of Sciences, Erciyes University, 38039 Kayseri, Turkey; e Department of Chemistry, Faculty of Science, Assiut University, 71515 Assiut, Egypt; f Kirkuk University, College of Science, Department of Chemistry, Kirkuk, Iraq

**Keywords:** crystal structure, pyrazolo­[3,4-*b*]pyrazine, hydrogen bonding, π–π inter­actions, scaffold compounds

## Abstract

In the title compound, C_15_H_15_N_7_, the phenyl ring is inclined by 19.86 (5)° to the mean plane of the pyrazolo­[3,4-*b*]pyrazine core. In the crystal, N—H⋯N and C—H⋯N hydrogen bonds form [010] chains, which stack *via* π–π inter­actions [centroid–centroid distance between the pyrazole rings = 3.4322 (7) Å].

## Related literature   

For the synthesis of similar pyrazolo­[3,4-*b*]pyrazines, see: El-Emary & El-Kashef (2013 ▶). For different biological and industrial applications of pyrazolo­pyrazine scaffold compounds, see: El-Emary *et al.* (1998 ▶); El-Kashef *et al.* (2000 ▶); El-Emary (2006 ▶); Rangnekar (1990 ▶).
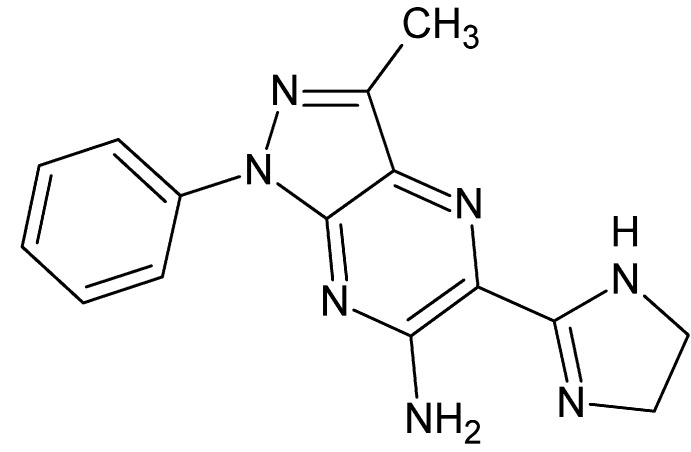


## Experimental   

### Crystal data  

C_15_H_15_N_7_
*M* *_r_* = 293.34
Monoclinic, 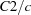

*a* = 7.9412 (2) Å
*b* = 15.7078 (3) Å
*c* = 22.3276 (4) Å
β = 98.573 (1)°
*V* = 2754.00 (10) Å^3^
*Z* = 8
Cu *K*α radiation
μ = 0.75 mm^−1^
*T* = 150 K
0.15 × 0.10 × 0.05 mm

### Data collection  

Bruker D8 VENTURE PHOTON 100 CMOS diffractometer
Absorption correction: multi-scan (*SADABS*; Bruker, 2014 ▶) *T*_min_ = 0.93, *T*_max_ = 0.97
21033 measured reflections
2685 independent reflections
2335 reflections with *I* > 2σ(*I*)
*R*_int_ = 0.031

### Refinement  

*R*[*F* ^2^ > 2σ(*F* ^2^)] = 0.035
*wR*(*F* ^2^) = 0.094
*S* = 1.04
2685 reflections
201 parameters
H-atom parameters constrained
Δρ_max_ = 0.23 e Å^−3^
Δρ_min_ = −0.17 e Å^−3^

### 

Data collection: *APEX2* (Bruker, 2014 ▶); cell refinement: *SAINT* (Bruker, 2014 ▶); data reduction: *SAINT*; program(s) used to solve structure: *SHELXT* (Sheldrick, 2008 ▶); program(s) used to refine structure: *SHELXL2014* (Sheldrick, 2008 ▶); molecular graphics: *DIAMOND* (Brandenburg & Putz, 2012 ▶); software used to prepare material for publication: *SHELXTL2014* (Sheldrick, 2008 ▶).

## Supplementary Material

Crystal structure: contains datablock(s) global, I. DOI: 10.1107/S160053681402354X/hg5415sup1.cif

Structure factors: contains datablock(s) I. DOI: 10.1107/S160053681402354X/hg5415Isup2.hkl

Supporting information file. DOI: 10.1107/S160053681402354X/hg5415Isup3.cml

**Fig. 1.**
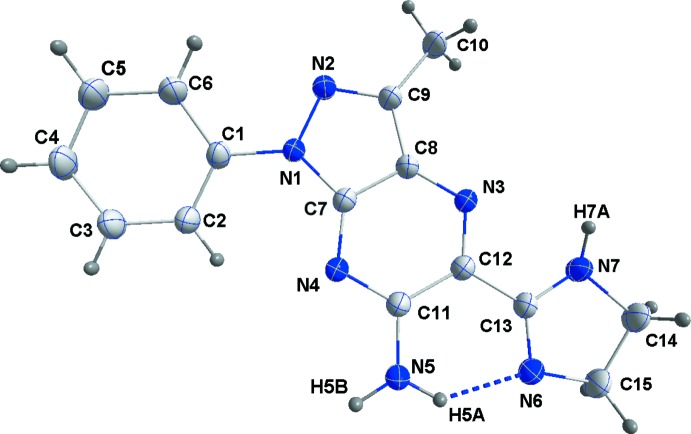
The title molecule with numbering scheme and 50% probability ellipsoids. The intramolecular hydrogen bond is shown as a blue dotted line.

. DOI: 10.1107/S160053681402354X/hg5415fig1.tif
The title mol­ecule with numbering scheme and 50% probability ellipsoids. The intra­molecular hydrogen bond is shown as a blue dotted line.

**Fig. 2.**
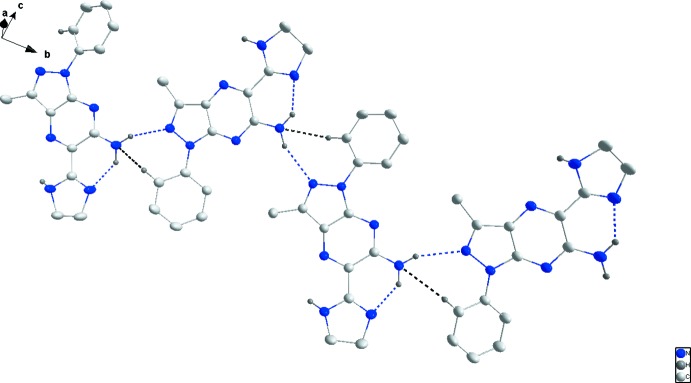
Plan view of the chain showing N—H···N and C—H···N hydrogen bonds as blue and black dotted lines, respectively.

. DOI: 10.1107/S160053681402354X/hg5415fig2.tif
Plan view of the chain showing N—H⋯N and C—H⋯N hydrogen bonds as blue and black dotted lines, respectively.

**Fig. 3.**
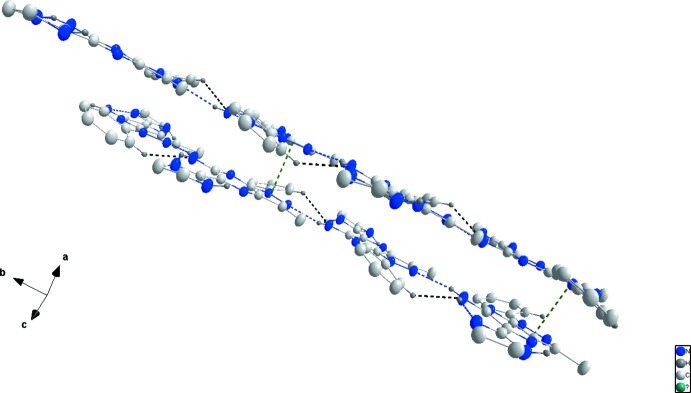
Elevation view of two chains showing the π-π interactions as green dotted lines.

**Fig. 4.**
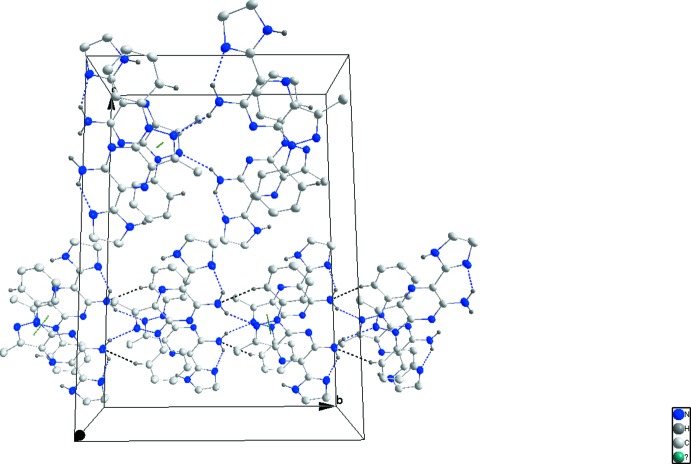
Packing viewed down the a axis.

. DOI: 10.1107/S160053681402354X/hg5415fig3.tif
Elevation view of two chains showing the π-π inter­actions as green dotted lines.

a . DOI: 10.1107/S160053681402354X/hg5415fig4.tif
Packing viewed down the *a* axis.

CCDC reference: 1031105

Additional supporting information:  crystallographic information; 3D view; checkCIF report

## Figures and Tables

**Table 1:** Hydrogen-bond geometry (, )

*D*H*A*	*D*H	H*A*	*D* *A*	*D*H*A*
C6H6N5^i^	0.95	2.56	3.3483(17)	140
N5H5*B*N2^ii^	0.91	2.31	3.1702(15)	158
N5H5*A*N6	0.91	1.97	2.7111(15)	138

Symmetry codes: (i) 
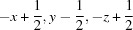
; (ii) 
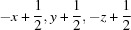
.

## supplementary crystallographic information

### S1. Comment

Pyrazine scaffold compounds are an interesting class of fused heterocyclic compounds due to their diverse properties in medicinal and applied chemistry. Pyrazolo[3,4-*b*]pyrazine derivatives have been reported to act as blood platelet aggregation inhibitors and bone metabolism improvers (El-Emary & El-Kashef, 2013). They also show antifungal and antiparasitic activities (El-Emary *et al.*, 1998; El-Kashef *et al.*, 2000; El-Emary, 2006). In addition, they are used as dye dispersants and as fluorescents (Rangnekar, 1990). In view of this medicinal importance, the crystal structure determination of the title compound was carried out and the results are presented here.

In the title compound, the pyrazolo[3,4-*b*]pyrazine core is planar (r.m.s. deviation 0.0089) with the phenyl ring inclined by 19.86 (5)° to it. An intramolecular N5—H5a···N6 hydrogen bond (Table 1 and Fig. 1) keeps the dihydroimidazolyl substituent nearly coplanar with the core. Intermolecular N5—H5b···N2 and C6—H6···N5 hydrogen bonds form chains of molecules (Table 1 and Fig. 2) which stack *via*π–π interactions between pyrazine rings (*Cg*^i^···*Cg*^ii^ = *Cg*^iii^··· *Cg*^iv^ = 3.43 Å. *Cg*^i^: 0.5 - *x*, 1/2 + *y*, 0.5 - *z*; *Cg*^ii^: -1/2 + *x*, 1/2 + *y*, *z*; *Cg*^iii^: 0.5 - *x*, -1/2 + *y*, 0.5 - *z*; *Cg*^iv^: -1/2 + *x*, -1/2 + *y*, *z*. Figs. 3 and 4). The chains run approximately parallel to (102).

### S2. Experimental

The title compound has been prepared according to our reported method (El-Kashef *et al.*, 2000). The product was crystallized from dioxan to furnish a good yield (68%) of colourles crystals (m.p. 503–505 K) which were of suffiecient quality for X-ray diffraction.

### S3. Refinement

H-atoms attached to carbon were placed in calculated positions (C—H = 0.95 - 0.98 Å) while those attached to nitrogen were placed in locations derived from a difference map and their parameters adjusted to give N—H = 0.91 Å. All were included as riding contributions with isotropic displacement parameters 1.2 - 1.5 times those of the attached atoms.

### Crystal data

**Table:**

C_15_H_15_N_7_	*F*(000) = 1232
*M**_r_* = 293.34	*D*_x_ = 1.415 Mg m^−^^3^
Monoclinic, *C*2/*c*	Cu *K*α radiation, λ = 1.54178 Å
*a* = 7.9412 (2) Å	Cell parameters from 9946 reflections
*b* = 15.7078 (3) Å	θ = 4.0–71.7°
*c* = 22.3276 (4) Å	µ = 0.75 mm^−^^1^
β = 98.573 (1)°	*T* = 150 K
*V* = 2754.00 (10) Å^3^	Block, colourless
*Z* = 8	0.15 × 0.10 × 0.05 mm

### Data collection

**Table:**

Bruker D8 VENTURE PHOTON 100 CMOS diffractometer	2685 independent reflections
Radiation source: INCOATEC IµS micro–focus source	2335 reflections with *I* > 2σ(*I*)
Mirror monochromator	*R*_int_ = 0.031
Detector resolution: 10.4167 pixels mm^-1^	θ_max_ = 72.0°, θ_min_ = 4.0°
ω scans	*h* = −9→9
Absorption correction: multi-scan (*SADABS*; Bruker, 2014)	*k* = −19→18
*T*_min_ = 0.93, *T*_max_ = 0.97	*l* = −25→27
21033 measured reflections	

### Refinement

**Table:**

Refinement on *F*^2^	Secondary atom site location: difference Fourier map
Least-squares matrix: full	Hydrogen site location: mixed
*R*[*F*^2^ > 2σ(*F*^2^)] = 0.035	H-atom parameters constrained
*wR*(*F*^2^) = 0.094	*w* = 1/[σ^2^(*F*_o_^2^) + (0.047*P*)^2^ + 1.7272*P*] where *P* = (*F*_o_^2^ + 2*F*_c_^2^)/3
*S* = 1.04	(Δ/σ)_max_ < 0.001
2685 reflections	Δρ_max_ = 0.23 e Å^−^^3^
201 parameters	Δρ_min_ = −0.17 e Å^−^^3^
0 restraints	Extinction correction: *SHELXL2014* (Sheldrick, 2008), Fc^*^=kFc[1+0.001xFc^2^λ^3^/sin(2θ)]^-1/4^
Primary atom site location: structure-invariant direct methods	Extinction coefficient: 0.00012 (3)

### Special details

**Table:**

Geometry. All e.s.d.'s (except the e.s.d. in the dihedral angle between two l.s. planes) are estimated using the full covariance matrix. The cell e.s.d.'s are taken into account individually in the estimation of e.s.d.'s in distances, angles and torsion angles; correlations between e.s.d.'s in cell parameters are only used when they are defined by crystal symmetry. An approximate (isotropic) treatment of cell e.s.d.'s is used for estimating e.s.d.'s involving l.s. planes.
Refinement. Refinement of *F*^2^ against ALL reflections. The weighted *R*-factor *wR* and goodness of fit *S* are based on *F*^2^, conventional *R*-factors *R* are based on *F*, with *F* set to zero for negative *F*^2^. The threshold expression of *F*^2^ > σ(*F*^2^) is used only for calculating *R*-factors(gt) *etc*. and is not relevant to the choice of reflections for refinement. *R*-factors based on *F*^2^ are statistically about twice as large as those based on *F*, and *R*- factors based on ALL data will be even larger. H-atoms attached to carbon were placed in calculated positions (C—H = 0.95 - 0.98 Å) while those attached to nitrogen were placed in locations derived from a difference map and their parameters adjusted to give N—H = 0.91 Å. All were included as riding contributions with isotropic displacement parameters 1.2 - 1.5 times those of the attached atoms.

### Fractional atomic coordinates and isotropic or equivalent isotropic displacement parameters (Å^2^)

**Table:**

	*x*	*y*	*z*	*U*_iso_*/*U*_eq_	
N1	0.21440 (13)	0.23938 (6)	0.22995 (4)	0.0252 (2)	
N2	0.24278 (14)	0.15429 (6)	0.24683 (5)	0.0284 (3)	
N3	0.49830 (13)	0.27132 (6)	0.36366 (5)	0.0255 (2)	
N4	0.31514 (13)	0.37679 (6)	0.27055 (4)	0.0258 (2)	
N5	0.43603 (15)	0.49390 (7)	0.32160 (5)	0.0350 (3)	
H5A	0.5051	0.5149	0.3545	0.042*	
H5B	0.3806	0.5295	0.2931	0.042*	
N6	0.62895 (15)	0.47203 (7)	0.43134 (5)	0.0345 (3)	
N7	0.69317 (15)	0.33776 (7)	0.46313 (5)	0.0339 (3)	
H7A	0.7265	0.2859	0.4511	0.041*	
C1	0.10360 (15)	0.25899 (8)	0.17597 (5)	0.0255 (3)	
C2	0.03232 (16)	0.33968 (8)	0.16704 (6)	0.0292 (3)	
H2	0.0600	0.3829	0.1967	0.035*	
C3	−0.07919 (17)	0.35656 (9)	0.11468 (6)	0.0325 (3)	
H3	−0.1269	0.4119	0.1083	0.039*	
C4	−0.12216 (18)	0.29404 (9)	0.07151 (6)	0.0366 (3)	
H4	−0.2016	0.3057	0.0363	0.044*	
C5	−0.04798 (19)	0.21408 (9)	0.08019 (6)	0.0382 (3)	
H5	−0.0757	0.1711	0.0504	0.046*	
C6	0.06600 (18)	0.19648 (8)	0.13184 (6)	0.0322 (3)	
H6	0.1183	0.1421	0.1371	0.039*	
C7	0.30685 (15)	0.29182 (8)	0.27108 (5)	0.0241 (3)	
C8	0.39641 (15)	0.23910 (8)	0.31557 (5)	0.0251 (3)	
C9	0.35066 (16)	0.15415 (8)	0.29763 (6)	0.0278 (3)	
C10	0.4086 (2)	0.07325 (9)	0.32887 (7)	0.0377 (3)	
H10A	0.3465	0.0253	0.3079	0.057*	
H10B	0.3866	0.0753	0.3709	0.057*	
H10C	0.5309	0.0659	0.3284	0.057*	
C11	0.41803 (16)	0.40912 (8)	0.31815 (5)	0.0260 (3)	
C12	0.50810 (15)	0.35545 (8)	0.36559 (5)	0.0253 (3)	
C13	0.61270 (16)	0.39176 (8)	0.41964 (5)	0.0263 (3)	
C14	0.80754 (19)	0.38977 (9)	0.50567 (6)	0.0366 (3)	
H14A	0.9268	0.3854	0.4980	0.044*	
H14B	0.8018	0.3736	0.5482	0.044*	
C15	0.73523 (19)	0.47864 (9)	0.49128 (6)	0.0380 (3)	
H15A	0.6658	0.4970	0.5223	0.046*	
H15B	0.8283	0.5203	0.4901	0.046*	

### Atomic displacement parameters (Å^2^)

**Table:**

	*U*^11^	*U*^22^	*U*^33^	*U*^12^	*U*^13^	*U*^23^
N1	0.0278 (5)	0.0214 (5)	0.0242 (5)	−0.0011 (4)	−0.0036 (4)	−0.0001 (4)
N2	0.0326 (6)	0.0213 (5)	0.0288 (6)	−0.0016 (4)	−0.0037 (4)	0.0005 (4)
N3	0.0263 (5)	0.0237 (5)	0.0250 (5)	−0.0011 (4)	−0.0008 (4)	−0.0008 (4)
N4	0.0269 (6)	0.0230 (5)	0.0256 (5)	−0.0006 (4)	−0.0019 (4)	−0.0005 (4)
N5	0.0429 (7)	0.0222 (5)	0.0345 (6)	0.0006 (5)	−0.0123 (5)	−0.0005 (4)
N6	0.0385 (6)	0.0279 (6)	0.0329 (6)	−0.0012 (5)	−0.0082 (5)	−0.0059 (5)
N7	0.0434 (7)	0.0270 (6)	0.0269 (6)	−0.0027 (5)	−0.0087 (5)	−0.0004 (4)
C1	0.0244 (6)	0.0276 (6)	0.0233 (6)	−0.0040 (5)	−0.0007 (5)	0.0017 (5)
C2	0.0300 (7)	0.0289 (7)	0.0269 (6)	0.0004 (5)	−0.0014 (5)	−0.0016 (5)
C3	0.0319 (7)	0.0319 (7)	0.0315 (7)	0.0023 (5)	−0.0024 (6)	0.0031 (5)
C4	0.0370 (7)	0.0396 (8)	0.0289 (7)	−0.0038 (6)	−0.0095 (6)	0.0032 (6)
C5	0.0465 (8)	0.0337 (7)	0.0301 (7)	−0.0065 (6)	−0.0088 (6)	−0.0033 (6)
C6	0.0387 (7)	0.0251 (6)	0.0300 (7)	−0.0030 (5)	−0.0035 (6)	−0.0002 (5)
C7	0.0232 (6)	0.0249 (6)	0.0233 (6)	−0.0013 (5)	0.0009 (5)	−0.0011 (5)
C8	0.0260 (6)	0.0233 (6)	0.0245 (6)	0.0000 (5)	−0.0010 (5)	0.0002 (5)
C9	0.0300 (7)	0.0246 (6)	0.0270 (6)	−0.0013 (5)	−0.0018 (5)	−0.0002 (5)
C10	0.0463 (8)	0.0254 (7)	0.0364 (7)	−0.0021 (6)	−0.0108 (6)	0.0029 (5)
C11	0.0261 (6)	0.0244 (6)	0.0262 (6)	0.0006 (5)	−0.0004 (5)	−0.0016 (5)
C12	0.0251 (6)	0.0243 (6)	0.0253 (6)	−0.0011 (5)	0.0005 (5)	−0.0016 (5)
C13	0.0259 (6)	0.0268 (6)	0.0252 (6)	−0.0011 (5)	0.0007 (5)	−0.0008 (5)
C14	0.0408 (8)	0.0371 (7)	0.0280 (7)	−0.0054 (6)	−0.0075 (6)	−0.0028 (6)
C15	0.0398 (8)	0.0359 (7)	0.0339 (7)	−0.0033 (6)	−0.0089 (6)	−0.0089 (6)

### Geometric parameters (Å, º)

**Table:**

N1—C7	1.3638 (15)	C3—C4	1.3826 (19)
N1—N2	1.3978 (14)	C3—H3	0.9500
N1—C1	1.4161 (16)	C4—C5	1.389 (2)
N2—C9	1.3158 (17)	C4—H4	0.9500
N3—C12	1.3241 (16)	C5—C6	1.3836 (19)
N3—C8	1.3435 (16)	C5—H5	0.9500
N4—C7	1.3365 (16)	C6—H6	0.9500
N4—C11	1.3401 (16)	C7—C8	1.4028 (17)
N5—C11	1.3404 (16)	C8—C9	1.4246 (17)
N5—H5A	0.9100	C9—C10	1.4891 (18)
N5—H5B	0.9099	C10—H10A	0.9800
N6—C13	1.2902 (17)	C10—H10B	0.9800
N6—C15	1.4755 (17)	C10—H10C	0.9800
N7—C13	1.3732 (16)	C11—C12	1.4554 (17)
N7—C14	1.4620 (17)	C12—C13	1.4741 (16)
N7—H7A	0.9099	C14—C15	1.525 (2)
C1—C2	1.3904 (18)	C14—H14A	0.9900
C1—C6	1.3916 (18)	C14—H14B	0.9900
C2—C3	1.3829 (18)	C15—H15A	0.9900
C2—H2	0.9500	C15—H15B	0.9900
			
C7—N1—N2	110.30 (10)	N3—C8—C7	121.67 (11)
C7—N1—C1	130.20 (10)	N3—C8—C9	132.50 (11)
N2—N1—C1	119.48 (10)	C7—C8—C9	105.83 (11)
C9—N2—N1	106.98 (10)	N2—C9—C8	110.29 (11)
C12—N3—C8	115.30 (10)	N2—C9—C10	121.41 (11)
C7—N4—C11	113.36 (10)	C8—C9—C10	128.29 (11)
C11—N5—H5A	116.9	C9—C10—H10A	109.5
C11—N5—H5B	122.3	C9—C10—H10B	109.5
H5A—N5—H5B	120.8	H10A—C10—H10B	109.5
C13—N6—C15	106.19 (11)	C9—C10—H10C	109.5
C13—N7—C14	107.03 (11)	H10A—C10—H10C	109.5
C13—N7—H7A	118.0	H10B—C10—H10C	109.5
C14—N7—H7A	120.9	N4—C11—N5	117.98 (11)
C2—C1—C6	120.12 (12)	N4—C11—C12	122.21 (11)
C2—C1—N1	120.56 (11)	N5—C11—C12	119.81 (11)
C6—C1—N1	119.31 (11)	N3—C12—C11	122.30 (11)
C3—C2—C1	119.46 (12)	N3—C12—C13	115.85 (11)
C3—C2—H2	120.3	C11—C12—C13	121.83 (11)
C1—C2—H2	120.3	N6—C13—N7	116.00 (11)
C4—C3—C2	120.89 (13)	N6—C13—C12	124.83 (11)
C4—C3—H3	119.6	N7—C13—C12	119.08 (11)
C2—C3—H3	119.6	N7—C14—C15	101.34 (10)
C3—C4—C5	119.33 (12)	N7—C14—H14A	111.5
C3—C4—H4	120.3	C15—C14—H14A	111.5
C5—C4—H4	120.3	N7—C14—H14B	111.5
C6—C5—C4	120.54 (13)	C15—C14—H14B	111.5
C6—C5—H5	119.7	H14A—C14—H14B	109.3
C4—C5—H5	119.7	N6—C15—C14	105.85 (10)
C5—C6—C1	119.60 (13)	N6—C15—H15A	110.6
C5—C6—H6	120.2	C14—C15—H15A	110.6
C1—C6—H6	120.2	N6—C15—H15B	110.6
N4—C7—N1	128.29 (11)	C14—C15—H15B	110.6
N4—C7—C8	125.12 (11)	H15A—C15—H15B	108.7
N1—C7—C8	106.59 (10)		
			
C7—N1—N2—C9	0.15 (14)	N1—N2—C9—C8	−0.06 (15)
C1—N1—N2—C9	179.34 (11)	N1—N2—C9—C10	179.61 (12)
C7—N1—C1—C2	−20.6 (2)	N3—C8—C9—N2	178.77 (13)
N2—N1—C1—C2	160.36 (11)	C7—C8—C9—N2	−0.05 (15)
C7—N1—C1—C6	159.58 (12)	N3—C8—C9—C10	−0.9 (2)
N2—N1—C1—C6	−19.42 (17)	C7—C8—C9—C10	−179.69 (14)
C6—C1—C2—C3	1.55 (19)	C7—N4—C11—N5	179.53 (11)
N1—C1—C2—C3	−178.23 (11)	C7—N4—C11—C12	−0.94 (17)
C1—C2—C3—C4	0.8 (2)	C8—N3—C12—C11	−1.44 (17)
C2—C3—C4—C5	−2.0 (2)	C8—N3—C12—C13	177.08 (10)
C3—C4—C5—C6	0.9 (2)	N4—C11—C12—N3	2.23 (19)
C4—C5—C6—C1	1.4 (2)	N5—C11—C12—N3	−178.24 (12)
C2—C1—C6—C5	−2.6 (2)	N4—C11—C12—C13	−176.20 (11)
N1—C1—C6—C5	177.17 (12)	N5—C11—C12—C13	3.32 (18)
C11—N4—C7—N1	179.70 (12)	C15—N6—C13—N7	0.00 (16)
C11—N4—C7—C8	−0.87 (18)	C15—N6—C13—C12	176.46 (12)
N2—N1—C7—N4	179.34 (12)	C14—N7—C13—N6	−12.26 (16)
C1—N1—C7—N4	0.3 (2)	C14—N7—C13—C12	171.07 (11)
N2—N1—C7—C8	−0.18 (13)	N3—C12—C13—N6	−175.55 (12)
C1—N1—C7—C8	−179.25 (12)	C11—C12—C13—N6	3.0 (2)
C12—N3—C8—C7	−0.33 (17)	N3—C12—C13—N7	0.80 (17)
C12—N3—C8—C9	−178.99 (13)	C11—C12—C13—N7	179.33 (11)
N4—C7—C8—N3	1.63 (19)	C13—N7—C14—C15	17.78 (15)
N1—C7—C8—N3	−178.84 (11)	C13—N6—C15—C14	11.67 (16)
N4—C7—C8—C9	−179.40 (12)	N7—C14—C15—N6	−17.76 (15)
N1—C7—C8—C9	0.14 (13)		

### Hydrogen-bond geometry (Å, º)

**Table:**

*D*—H···*A*	*D*—H	H···*A*	*D*···*A*	*D*—H···*A*
C6—H6···N5^i^	0.95	2.56	3.3483 (17)	140
N5—H5*B*···N2^ii^	0.91	2.31	3.1702 (15)	158
N5—H5*A*···N6	0.91	1.97	2.7111 (15)	138

Symmetry codes: (i) −*x*+1/2, *y*−1/2, −*z*+1/2; (ii) −*x*+1/2, *y*+1/2, −*z*+1/2.
