# Supplementary figures and images for: Crystal structure of 5-(4,5-di­hydro-1H-imidazol-2-yl)-3-methyl-1-phenyl-1H-pyrazolo­[3,4-b]pyrazin-6-amine
Source: Acta Crystallogr Sect E Struct Rep Online. 2014 Oct 31;70(Pt 11):o1212–3. doi: 10.1107/S160053681402354X (PMC4257272; doi:10.1107/S160053681402354X)

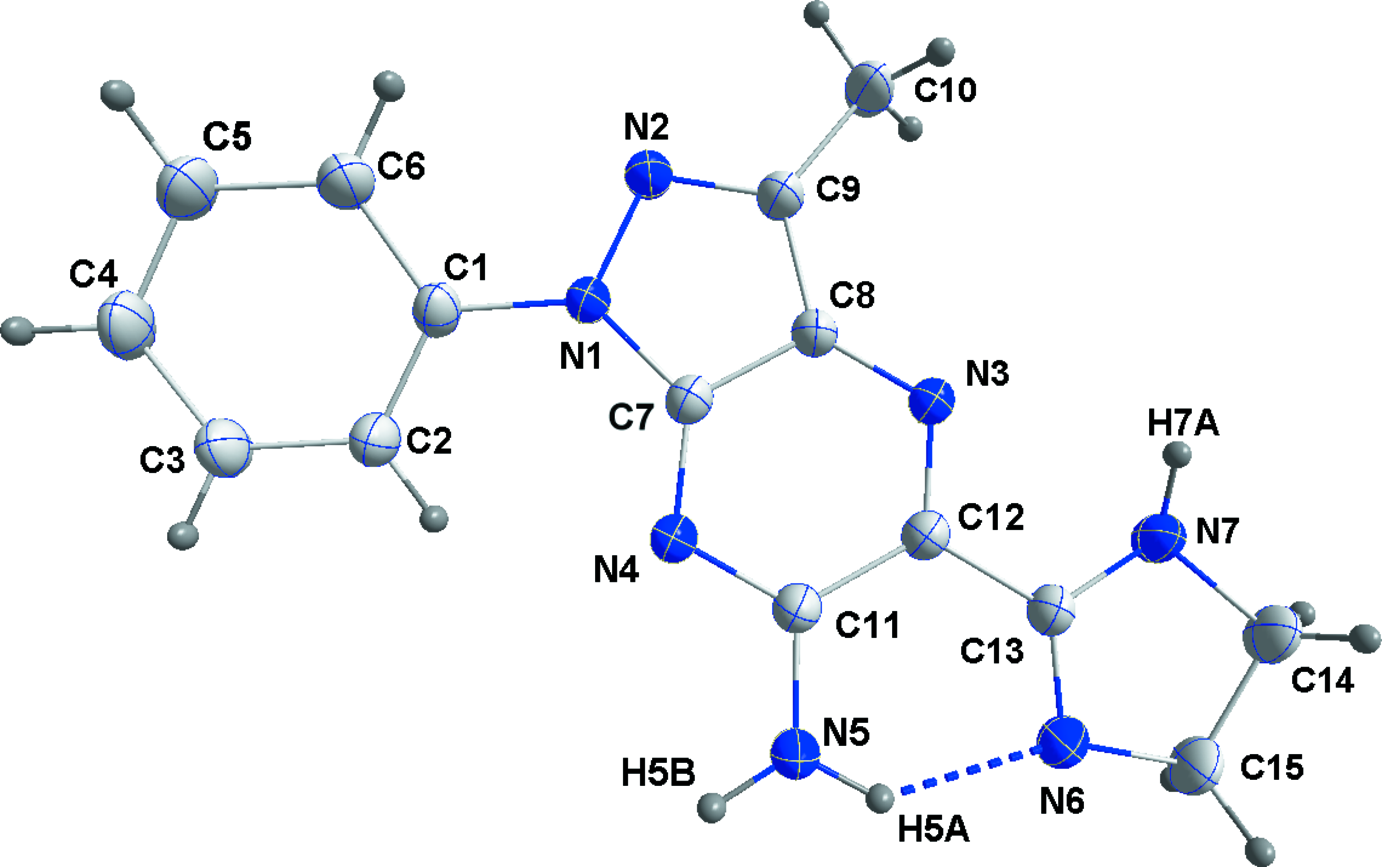

Supplement: Supplementary file 4 [file e-70-o1212-fig1.tif]

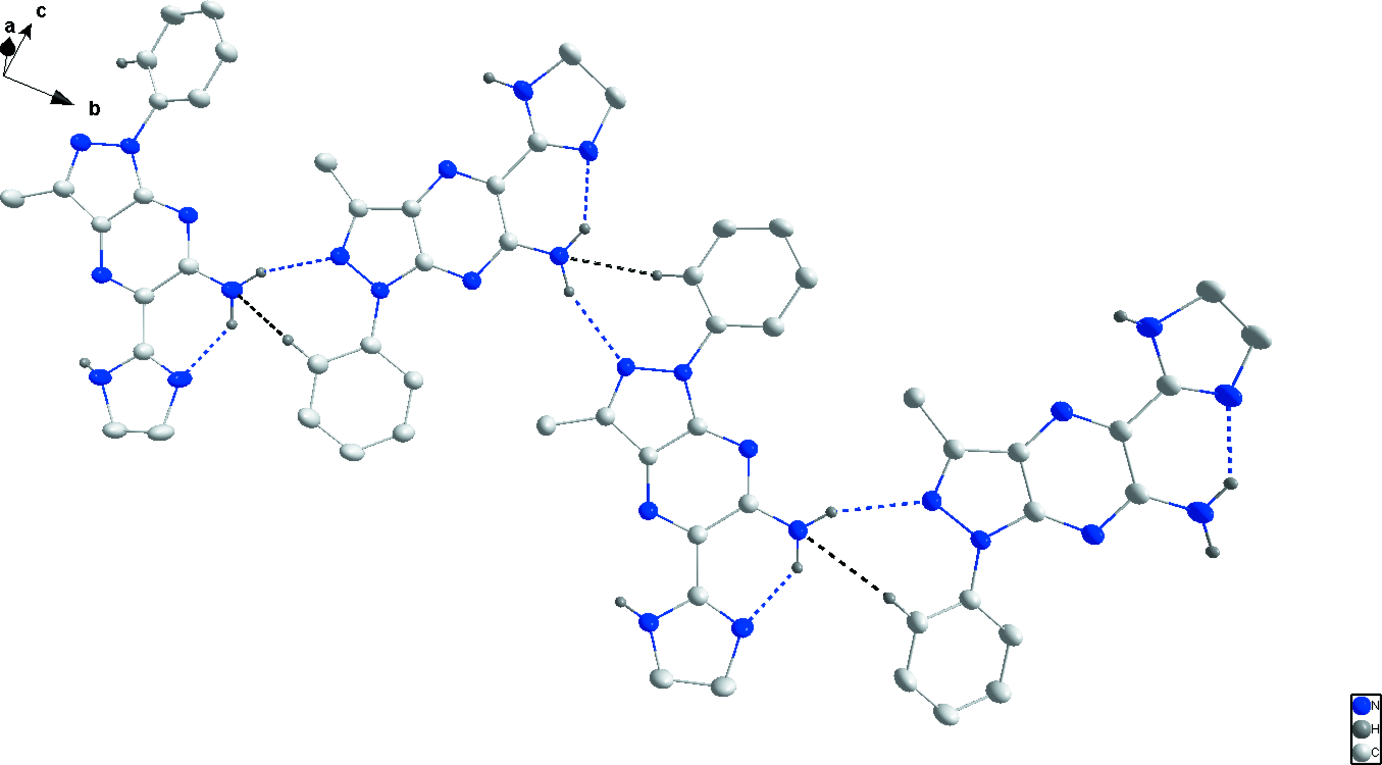

Supplement: Supplementary file 5 [file e-70-o1212-fig2.tif]

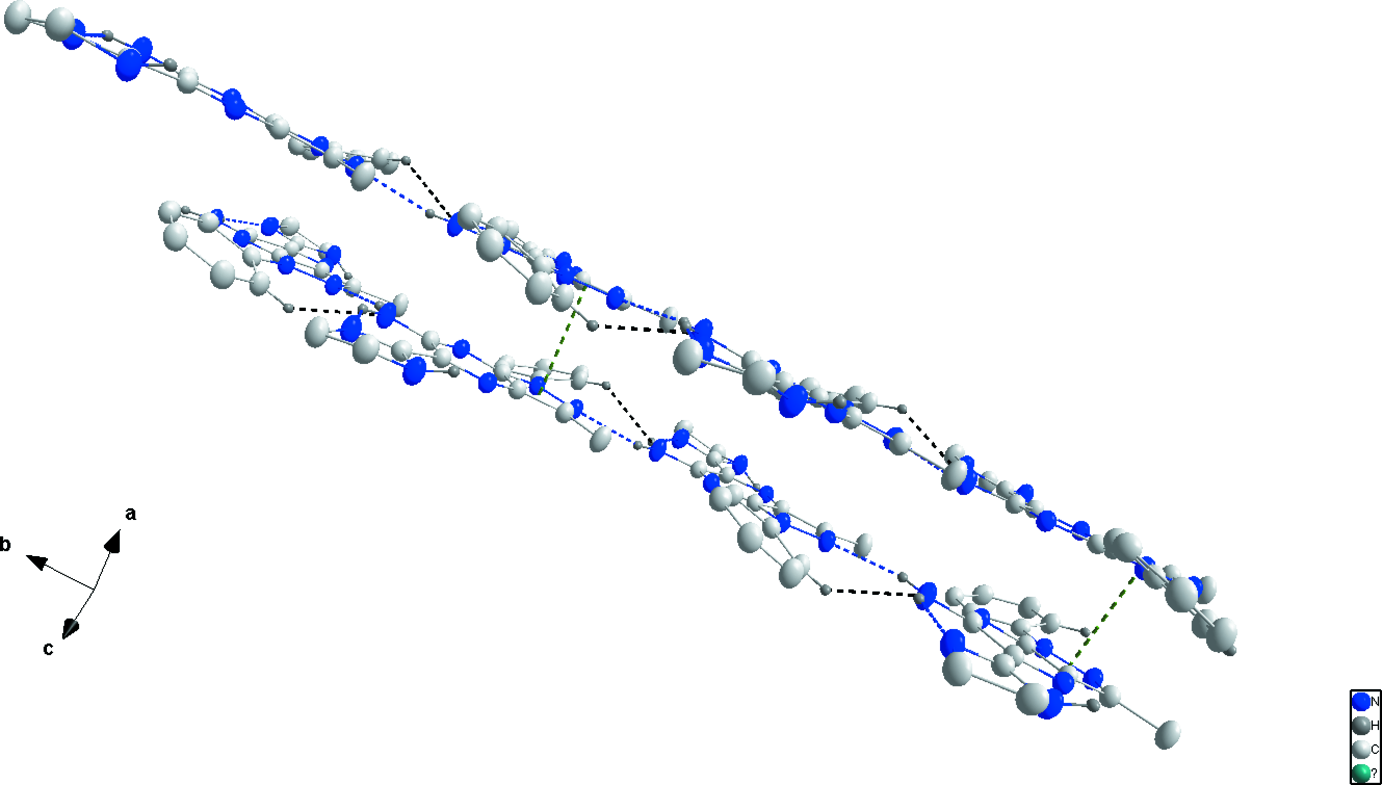

Supplement: Supplementary file 6 [file e-70-o1212-fig3.tif]

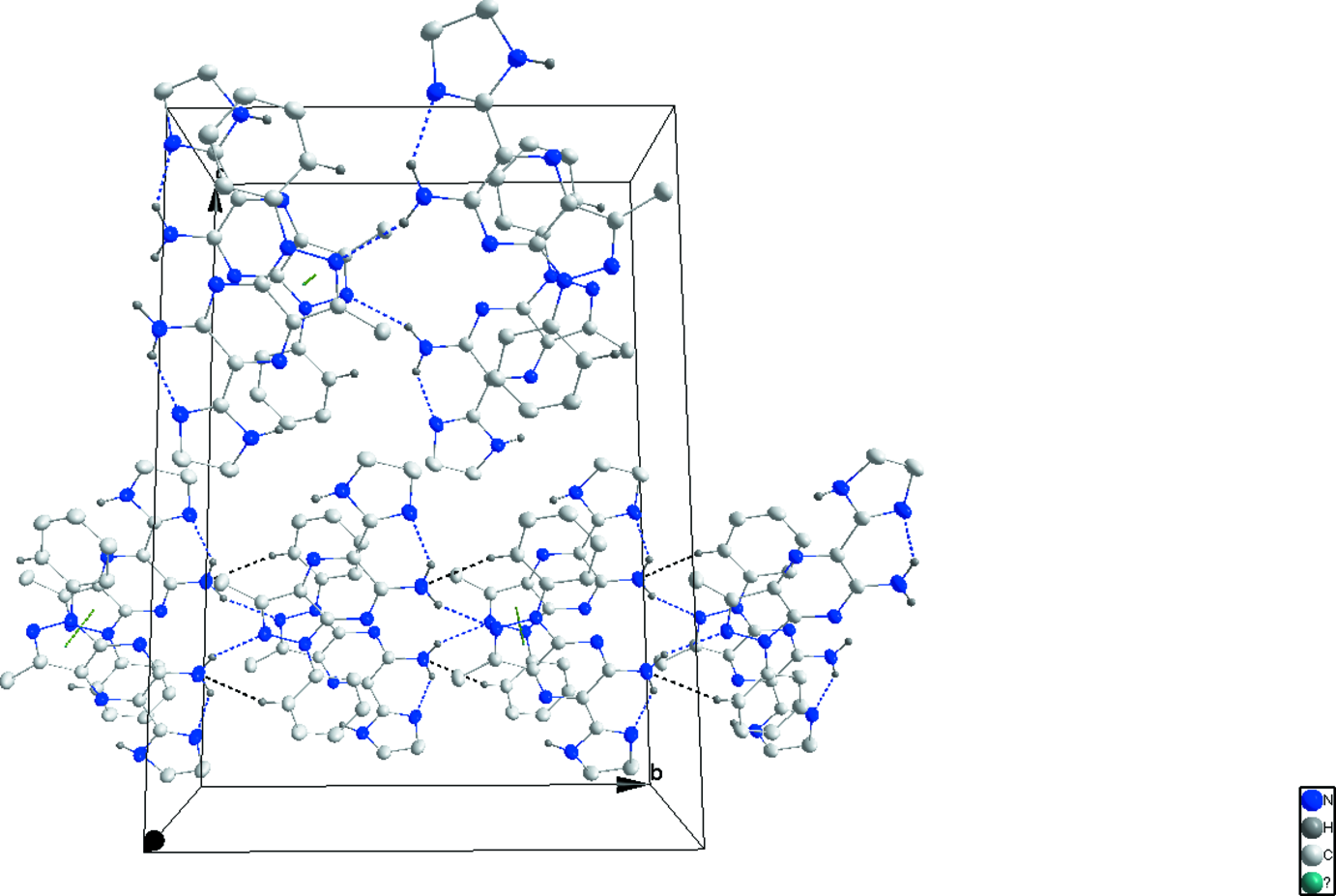

Supplement: Supplementary file 7 [file e-70-o1212-fig4.tif]
